# Disruption of Timing: NeuroHIV Progression in the Post-cART Era

**DOI:** 10.1038/s41598-018-36822-1

**Published:** 2019-01-29

**Authors:** Kristen A. McLaurin, Hailong Li, Rosemarie M. Booze, Charles F. Mactutus

**Affiliations:** Program in Behavioral Neuroscience, Department of Psychology, Barnwell College, 1512 Pendleton Street, University of South Carolina, Columbia, SC 29208 USA

## Abstract

The marked increase in life expectancy for HIV-1 seropositive individuals, following the great success of combination antiretroviral therapy (cART), heralds an examination of the progression of HIV-1 associated neurocognitive disorders (HAND). However, since the seminal call for animal models of HIV-1/AIDS in 1988, there has been no extant *in vivo* animal model system available to provide a truly longitudinal study of HAND. Here, we demonstrate that the HIV-1 transgenic (Tg) rat, resembling HIV-1 seropositive individuals on lifelong cART, exhibits age-related, progressive neurocognitive impairments (NCI), including alterations in learning, sustained attention, flexibility, and inhibition; deficits commonly observed in HIV-1 seropositive individuals. Pyramidal neurons from layers II-III of the medial prefrontal cortex (mPFC) displayed profound synaptic dysfunction in HIV-1 Tg animals relative to controls; dysfunction that was characterized by alterations in dendritic branching complexity, synaptic connectivity, and dendritic spine morphology. NCI and synaptic dysfunction in pyramidal neurons from layers II-III of the mPFC independently identified the presence of the HIV-1 transgene with at least 78.5% accuracy. Thus, even in the absence of sensory or motor system deficits and comorbidities, HAND is a neurodegenerative disease characterized by age-related disease progression; impairments which may be due, at least partly, to synaptic dysfunction in the mPFC. Further, the progression of HAND with age in the HIV-1 Tg rat and associated synaptic dysfunction affords an instrumental model system for the development of therapeutics and functional cure strategies.

## Introduction

Human immunodeficiency virus type 1 (HIV-1) remains a human health pandemic, with approximately 36.7 million individuals living with the disease worldwide^1^. Older individuals (>50 years of age) account for approximately 30–50% of HIV-1 seropositive individuals in high-resource countries^2^ following the great success of combination antiretroviral therapy (cART); a prevalence that is expected to reach approximately 73% by 2030^3^. In the post-cART era, milder forms of neurocognitive impairment (NCI) have become a hallmark of HIV-1, afflicting between 40–70% of HIV-1 seropositive individuals^4–6^. Cross-sectional studies have provided a wealth of knowledge on HIV-1 associated neurocognitive disorders (HAND), characterized by deficits in higher-order cognitive processes [e.g., attention, working memory, executive function^6,7^]. However, extrapolating cross-sectional findings to age-related disease progression is inferentially fraught^8^, heralding an examination of the progression of HAND using a longitudinal experimental design.

Since the seminal call for animal models of HIV-1/AIDS^9^, there has been no extant *in vivo* animal model system available to provide a truly longitudinal study of HAND. The HIV-1 transgenic (Tg) rat, originally developed by Reid *et al*.^10^, contains a *gag*-*pol* deleted provirus regulated by the human viral promoter. HIV-1 Tg rats exhibit general good health through advancing age, displaying steady growth rates^11–13^, intact sensory (i.e., auditory, visual, gustatory) and motor system function^11,14^, and a lifespan of approximately 21 months of age^11^. Cross-sectional studies have critically tested the utility of the HIV-1 Tg rat as a model for NCI; multiple laboratories have reported alterations in temporal processing [e.g.^13^], sustained attention [e.g.^15^], learning^15,16^, and memory^17^. A longitudinal assessment of NCI for the progression of HAND, and associated neural mechanisms, in the HIV-1 Tg rat, however, remains a critical knowledge gap.

Thus, in the present study, the HIV-1 Tg rat was used to establish the trajectory of NCI, in the absence of sensory or motor system deficits^14^ and comorbidities, across the functional lifespan. It was hypothesized that HIV-1 Tg rats, relative to F344/N controls, would display progressive, sex-dependent expression of NCI across the function lifespan; impairments that were associated with synaptic dysfunction in pyramidal neurons from layers II-III of the medial prefrontal cortex (mPFC) and/or neuroinflammatory markers in the hippocampus. A longitudinal experimental design was used to assess multiple neurocognitive domains, including learning, sustained attention, flexibility and inhibition, from approximately postnatal day (PD) 60 through PD 600. Associated neural mechanisms, including synaptic dysfunction in the mPFC and putative markers of neuroinflammation in the hippocampus were also evaluated. The progression of HAND with age in the HIV-1 Tg rat and associated synaptic dysfunction in the mPFC affords an instrumental model system for the development of therapeutics and functional cure strategies.

## Methods

### Experimental Design

**Figure 1:**
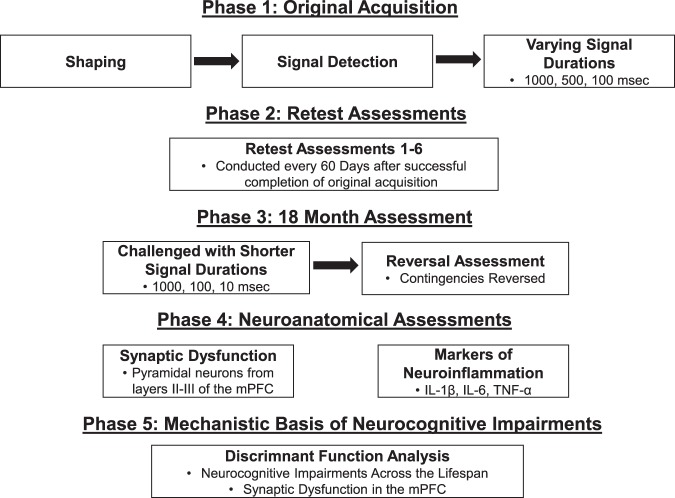
Schematic of the experimental design.

The experimental design for all neurocognitive assessments and neuroanatomical assessments is illustrated in Fig. 1.

### Animals

Across 12 months, Fischer (F344/N; Harlan Laboratories Inc., Indianapolis, IN) HIV-1 Tg (*N* = 20 litters) and control (*N* = 17 litters) rats were received at the animal vivarium, between PD 7 and PD 9, housed with their biological dam. At approximately PD 21, animals were sampled from each litter (HIV-1 Tg: male, *n* = 37, female, *n* = 33; Control: male *n* = 34; female, *n* = 33), weaned and pair- or group-housed with animals of the same sex for the duration of experimentation.

During the original acquisition of signal detection, animals were placed on food restriction, beginning at approximately PD 60, to maintain 85% body weight. After animals successfully acquired the signal detection task (PD 100-PD 277), rodent food was again provided *ad libitum*, including during all retest assessments. HIV-1 Tg and control animals had *ad libitum* access to water throughout the duration of the study.

HIV-1 Tg and control animals were sacrificed following the successful completion of the reversal task at approximately 20 months of age to assess synaptic dysfunction in pyramidal neurons from layers II-III of the mPFC and putative markers of neuroinflammation in the hippocampus. Due to health issues, including significant weight loss (*n* = 23), tumors (*n* = 6), natural death (*n* = 3) or other (*n* = 4), some animals were euthanized prior to 20 months of age.

Recommendations in the Guide for the Care and Use of Laboratory Animals of the National Institutes of Health were used for the maintenance of HIV-1 Tg and control animals in AAALAC-accredited facilities. The targeted environmental conditions for the animal vivarium were 21° ± 2°C, 50% ± 10% relative humidity and a 12-h light:12-h dark cycle with lights on at 0700 h (EST). The Institutional Animal Care and Use Committee (IACUC) at the University of South Carolina approved the project protocol (Federal Assurance # D16-00028).

### Phase 1: Original Acquisition

#### Apparatus

Sixteen operant chambers, located inside sound-attenuating chambers (Med Associates, Inc., Fairfax, VT), were used to train animals in a signal detection task tapping sustained attention. A pellet dispenser (45 mg), located between two retractable levers, and three panel lights were located on the front wall of the operant chambers. Only the panel light located above the pellet dispenser was used in the present experiment. A house light was located at the top of the rear wall of the operant chamber. The house light, used for shaping, and central panel light, used for signal presentation during the original acquisition of signal detection and retest assessments, were incandescent (22 lux). PC and Med-PC for Windows software (V 4.1.3; Med Associates, Inc., Fairfax, VT) controlled signal presentation, lever operation, reinforcement delivery, and data collection.

#### Shaping

A standard shaping response protocol was used to train animals to lever-press at approximately 2 months of age, described in detail by McLaurin *et al*.^18^. All animals met criteria in shaping (i.e., 60 reinforcers for 3 consecutive or 5 non-consecutive days), indicating learning the operant response, prior to beginning the signal detection task.

#### Signal Detection

Following successful acquisition of shaping, all HIV-1 Tg (*N* = 20 litters; male, *n* = 37, female, *n* = 33) and control animals (*N* = 17 litters; male *n* = 34; female, *n* = 33) were trained in a signal detection task, tapping sustained attention.

Three vigilance programs, initially described by McGaughy and Sarter^19^, were employed. In brief, animals were trained to discriminate between signal (i.e., central panel light illumination) and non-signal (i.e., no illumination) trials. In the first vigilance program, consisting of 160 trials per session, termination of the stimulus light was contingent upon a response. In the second vigilance program, consisting of 160 trials per session, the length of the stimulus light was 1 sec. Correction trials, including up to three repetitions of the trial, and force-choice trials were an integral component of the first two vigilance programs, and occurred when an animal responded incorrectly. In the third vigilance program, consisting of 162 trials per session, the length of the stimulus was manipulated (i.e., 1000, 500, 100 msec) across trials using a block randomized experimental design. Correction trials and force-choice trials were removed in the third vigilance program. During the initial acquisition, no data was censored i.e., each and every animal successfully acquired the signal detection task. A preliminary report of original acquisition, on approximately half of the animals, was presented in McLaurin *et al*.^18^. Additional methodological details are presented in the Supplementary Information Text available online.

### Phase 2: Retest Assessments

#### Apparatus

Throughout the retest assessments, HIV-1 Tg and control animals were assessed in the operant chambers described above.

#### Procedure

After the successful completion of original acquisition, HIV-1 Tg and control animals were assessed in the third vigilance program with varying signal durations every 60 days through approximately 18 months of age. Animals were given up to 60 days to successfully complete each retest assessment and were assessed in up to 6 retest assessments.

### Phase 3: 18 Month Assessment

#### Apparatus

At the 18 Month Assessment, HIV-1 Tg and control animals were assessed in the operant chambers described above with a minor modification. An LED light bulb (11 lux), instead of an incandescent light bulb, was used for signal presentation during the 18 month acquisition and reversal assessment due to the short (i.e., 10 msec) signal duration times.

#### Procedure

After the successful completion of the 6^th^ retest assessment or at 18 months of age, HIV-1 Tg and control animals were challenged with two tasks. First, HIV-1 Tg (*N* = 20 litters; male, *n* = 29, female, *n* = 29) and control animals (*N* = 17 litters; male *n* = 28; female, *n* = 31) were challenged for 5-consecutive days with shorter signal durations (i.e., 1000, 100, 10 msec). Second, HIV-1 Tg (*N* = 20 litters; male, *n* = 18, female, *n* = 29) and control animals (*N* = 17 litters; male *n* = 27; female, *n* = 28) were assessed in a reversal task, tapping flexibility and inhibition. Response contingencies were reversed from those originally learned in the signal detection task. Animals were given up to 60 days to successfully complete the reversal assessment or were sacrificed at approximately 20 months of age after 45 days in the task.

### Phase 4: Neuroanatomical Assessments

#### Synaptic Dysfunction

Preparation of Tissue: Animals were deeply anesthetized using sevoflurane (Abbot Laboratories, North Chicago, IL) and transcardially perfused using methodology adapted from Roscoe *et al*.^12^.

DiOlistic Labeling: A DiOlisitc labeling technique, originally described by Seabold *et al*.^20^, was used to visualize pyramidal neurons from layers II-III of the medial prefrontal cortex (mPFC). Methodology for the preparation of DiOlistic cartridges, preparation of Tefzel tubing, and DiOlistic labeling is described in detailed by McLaurin *et al*.^21^.

Pyramidal Neuron Dendritic Analysis and Spine Quantification: Pyramidal neurons from layers II-III of the mPFC, located approximately 3.7 mm to 2.2 mm anterior to Bregma^22^, were analyzed. Z-stack images were obtained using methodology previously reported^12^ on three to four pyramidal neurons from each animal [Control: *N* = 17 litters, male, *n* = 31, female, *n* = 30; HIV-1 Tg: *N* = 20 litters, male, *n* = 35, female, *n* = 33].

The AutoNeuron and AutoSpine extension modules, available in Neurolucida 360 (MicroBrightfield, Williston, VT), were used for the analysis of spine parameters. Selection criteria, including continuous dendritic staining, low background/dye clusters, and minimal diffusion of the DiI dye into the extracellular space, were used for spine analysis. Based on the selection criteria, one neuron from each animal was chosen for the analysis of spine parameters. Neurons not meeting the selection criteria were not included in the analysis, yielding Control, *N* = 16 litters, male, *n* = 2*6*, female, *n* = 20, and HIV-1 Tg *N* = 19 litters, male, *n* = 2*8*, female, *n* = 27.

Spine Parameters: An observer-assisted automatic classification of dendritic spines (i.e., thin, mushroom, stubby) was conducted using an algorithm in Neurolucida 360^23^. The number of segments at each branch order, an assessment of dendritic branching complexity, was examined for branch orders 1 through 10. A Sholl analysis was conducted to evaluate neuronal complexity (i.e., the number of intersections at each successive radii) and dendritic spine connectivity (i.e., the number of dendritic spines between each successive radii). Some animals were excluded due to processing errors, yielding Control, *N* = 16 litters, male, *n* = 21, female, *n* = 18, and HIV-1 Tg *N* = 17 litters, male, *n* = *26*, female, *n* = 23. Subsequently, dendritic spine morphology was assessed using three parameters, including backbone length (µm), head diameter (µm), and volume (µm^3^). Spine parameters were defined using well-accepted previously published results [i.e., backbone length, 0.1 to 4.1 µm^24^; head diameter, 0 to 0.825 µm^25^; volume, 0.05 to 0.5 µm^3^ ^26^].

#### Neuroinflammatory Markers

The selection of the hippocampus for the assessment of neuroinflammation was based on the publication of Fitting *et al*.^27,28^, which displayed frank cell loss in the hippocampus from viral protein exposure, maximizing the likelihood of detecting neuroinflammation.

Total RNA isolation and cDNA synthesis: Total RNA was isolated from 30 mg of hippocampal tissue using RNeasy FFPE kit (QIAGEN) according to manufacturer’s protocol (Control: *N* = 17 litters, male, *n* = 31 female, *n* = 30; HIV-1 Tg: *N* = 20 litters, male *n* = 32, female *n* = 30). The total RNA quality and quantity were assessed by a Nano drop spectrophotometer (Thermo Scientific, USA). One µg of total RNA from each sample was converted into cDNA by Cloned AMV first-strand cDNA synthesis kit (Invitrogen) for real-time PCR. The 20 μl first-strand cDNA synthesis reaction mixture contained 1 μl random-hexamer primer, 1 µg total RNA sample, 2 μl of 10 mM dNTP mix, 5x cDNA synthesis buffer, 1 μl of 0.1 M DTT, 1 μl RNaseOUT, 1 µl cloned AMV RT and 1 µl DEPC-treated water. The following conditions were used: 65 °C for 5 min, 25 °C for 10 min, 50 °C for 50 min and terminated at 85 °C for 5 min. The cDNA products were used immediately for further analysis.

Multiplex PCR (mPCR): Initially, hippocampal samples were probed for the expression of TNF-α, IL-1β, IL-6, NFκ-β and Iκ-β, and the internal control, GAPDH. Cytokine primers, a PCR reaction buffer containing dNTPs and a positive control were provided in a commercial mPCR kit from Maxibio Company (Rat Inflammatory Cytokines–Set 2, Cat. No. MP-70207, San Francisco, CA, as previously employed by Moran *et al*.^29^). However, there was no significant expression of TNF-α, IL-1β, IL-6, NFκ-β or Iκ-β in samples from HIV-1 Tg or F344/N control animals. Accordingly, we pursued analysis via real-time PCR.

Real-time PCR: The neuroinflammation related cytokines (TNF-α, IL-1β, IL-6) were quantified using a real-time PCR detection system and SsoAdvanced Universal SYBR Green Supermix kit (BIO-RAD). In brief, the 20 μl reaction mixture contained 10 μl 2x SsoAdvanced universal SYBR Green supermix, 1 μl forward primer and reverse primers (250 nM each), 1 μl template (100 ng) and 7 μl of DEPC-treated water. Reactions were performed with the DNA Engine Opticon 2 system (M J Research, USA) using the following cycling conditions: 30 sec at 95 °C and 40 cycles of 15 sec at 95 °C and 30 sec at 58 °C. Animals failing to reach threshold after 40 cycles were considered to have undetectable gene expression and accordingly these censored data were not included in the figures or statistical analysis, yielding Control, *N* = 8–15 litters, male, *n* = 7–18, female, *n* = 8–12, and HIV-1 Tg *N* = 11–13 litters, male, *n* = 6–14, female, *n* = 9–15, dependent upon gene. The data were analyzed using Intuitive Opticon Monitor TM software. Each primer, including the corresponding GenBank numbers, are listed in Supplementary Table 1, and β-Actin was used as an internal control. The relative gene expression of each neuroinflammatory marker (i.e., TNF-α, IL-1β, IL-6) was analyzed using the 2^−ΔΔCt^ method to examine relative changes in expression dependent upon genotype and biological sex^30^.

### Statistical Analysis

Given the nested experimental design (i.e., pups within litter), figures, created using GraphPad Prism 5 (GraphPad Software, Inc., La Jolla, CA), represent litter means and standard errors, dependent upon biological sex^31,32^.

Analysis of variance (ANOVA) and regression techniques were used for the analysis of all data (SAS/STAT Software 9.4, SAS Institute, Inc., Cary, NC; SPSS Statistics 24, IBM Corp., Somer, NY; GraphPad Software, Inc., La Jolla, CA). For all statistical analyses, litter was used as the unit of analysis to preclude the violation of the assumption of independence, leading to an inflation of Type 1 error rate^33^. Orthogonal decomposition of the repeated-measures factors or the conservative Greenhouse-Geisser *df* correction factor [*p*_GG_^34^] were used to preclude potential violations of sphericity. Effect size was assessed using partial eta squared (η_p_^2^). An alpha value of *p* ≤ 0.05 was considered significant for all statistical tests. Additional details on the statistical analyses are available in the Supplementary Information Text.

## Results

### Phase 1: Original Acquisition

#### The factor of biological sex was the driving factor for observed differences in the temporal process of acquisition and sustained attention during the initial acquisition of signal detection

**Figure 2:**
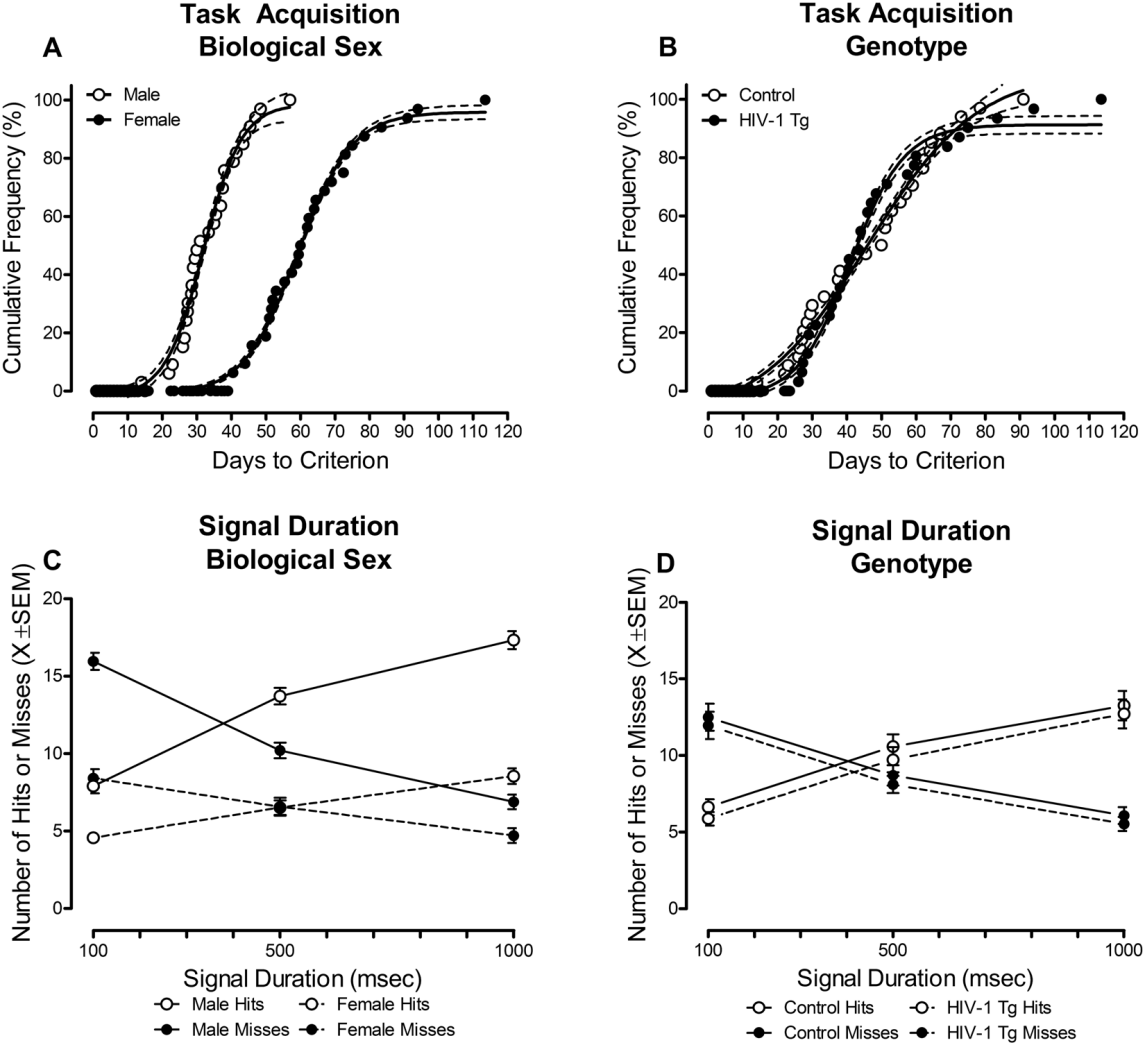
Initial acquisition of signal detection. (**A**) Assessment of the temporal process of acquisition revealed a main effect of sex (*p* ≤ 0.001), with female animals, independent of genotype, acquiring the signal detection operant task significantly slower than male animals. (**B**) Genotype failed to alter the temporal process of acquisition during the original acquisition phase (*p* > 0.05). (**C**) Assessment of sustained attention during the initial acquisition of signal detection revealed that female animals displayed a decreased response rate, as well as a prominent rightward shift in the loss of signal detection (i.e., the intersection of hits and misses; *p* ≤ 0.001) relative to male animals. (**D**) Presence of the HIV-1 transgene failed to alter sustained attention during initial acquisition (*p* > 0.05). Data are presented as cumulative frequencies with 95% confidence intervals fit to the curve (**A**,**B**) or mean ± SEM (**C**,**D**).

Temporal Process of Acquisition: All HIV-1 Tg and control animals were able to successfully acquire the task, achieving 70% accuracy for 5 consecutive or 7 non-consecutive days in each of the three vigilance programs. Profound sex differences were observed in the temporal process of task acquisition, best fit using a sigmoidal dose-response curve (Fig. 2A; *R*^2^s ≥ 0.98). Female animals, independent of genotype, acquired the task significantly slower than male animals (Main Effect: Sex [*F*(1,61) = 101.1, *p* ≤ 0.001]). Presence of the HIV-1 transgene failed to significantly alter the temporal process of initial acquisition of signal detection (Fig. 2B; *p* > 0.05).

Signal Detection: The factor of biological sex (Fig. 2C) was the driving factor for observed differences in sustained attention during the initial acquisition of signal detection. Female animals, collapsed across genotype, displayed a decreased response rate, as well as a prominent rightward shift in the loss of signal detection (i.e., where the number of hits and misses intersect), relative to male animals (approximately 535 msec vs. 436 msec, respectively; Sex × Response Type × Duration Interaction [*F*(2,122) = 74.0, *p*_GG_ ≤ 0.0001, η_p_^2^ = 0.548, Power = 1.0]) with a prominent linear-linear component [*F*(1,61) = 87.2, *p* ≤ 0.0001, η_p_^2^ = 0.588, Power = 1.0]. In sharp contrast, presence of the HIV-1 transgene failed to alter signal detection during initial acquisition (Fig. 2D; Genotype × Response Type × Duration Interaction [*p* > 0.05]).

### Phase 2: Retest Assessments

#### HIV-1 Tg animals, independent of biological sex, exhibited a progressive impairment in task acquisition across retest assessment

**Figure 3:**
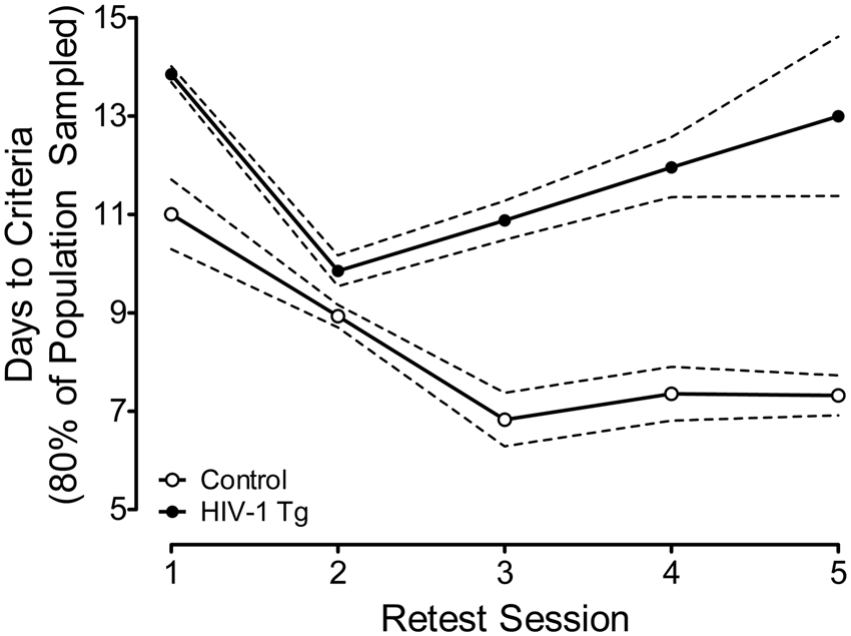
The number of days at which 80% of the population sampled met criteria (±95% Confidence Intervals (CI)) is illustrated as a function of genotype (HIV-1 Tg vs. control) and retest session. Points and 95% CI were derived from the cumulative frequency curves presented in Supplementary Fig. 1. Control animals exhibited an improvement in performance as a function of retest assessment, reaching asymptotic performance at approximately 7 days. In sharp contrast, HIV-1 Tg animals exhibited an initial improvement through retest assessment 2, followed by a progressive decline.

Days to Criteria: HIV-1 Tg and control animals were retested every 60 days in the third vigilance program, which manipulated signal duration length (1000, 500, 100 msec). Completion of the retest assessment required animals to meet criteria of 70% accuracy for 5 consecutive or 7 non-consecutive days. To assess the progression of task acquisition across retest assessments, the number of days required for 80% of the population sampled to meet criteria was quantified (Fig. 3). Control animals displayed a continuous improvement in task acquisition across retest assessments, reaching asymptotic performance at approximately 7 days. In sharp contrast, HIV-1 Tg animals exhibited an improvement in task acquisition through retest session 2, followed by a progressive decline. Assessment of the temporal process of acquisition at each individual retest assessment (see Supplementary Fig. 1) revealed a significant main effect of genotype (*p* ≤ 0.05) at all retest assessments.

#### Despite the “savings” afforded by repeated testing, HIV-1 Tg animals displayed a progressive, relative impairment in the detection of shorter signal durations

**Figure 4:**
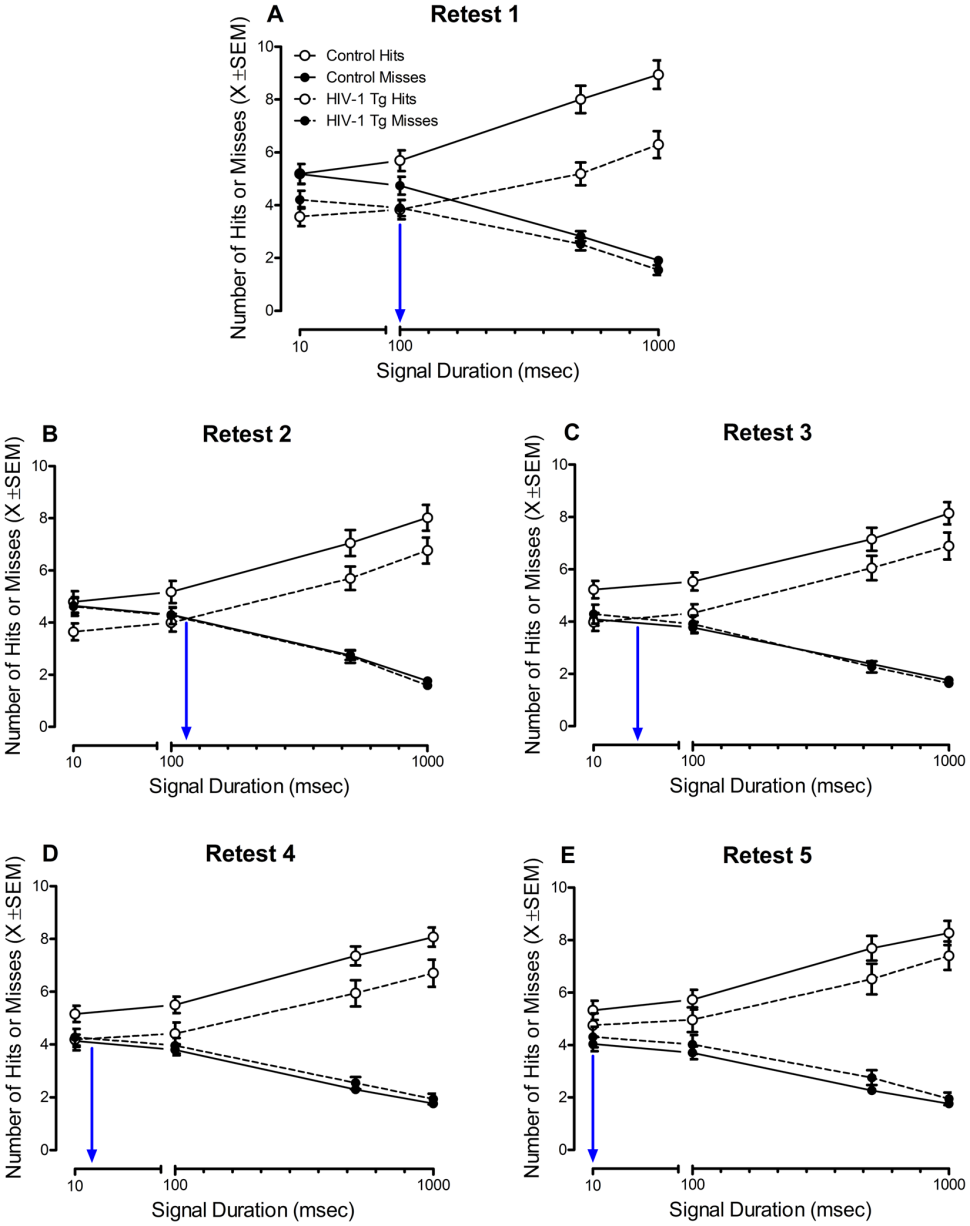
Sustained attention as a function of retest assessment. At each individual retest session (i.e, Retest 1: (**A**), Retest 2: (**B**), Retest 3: (**C**), Retest 4: (**D**), Retest 5: (**E**)) HIV-1 Tg animals, independent of biological sex, displayed an impairment in the detection of shorter signal durations (1000, 500, 100 msec). Across retest sessions, HIV-1 Tg animals displayed a progressive, relative impairment in sustained attention (*p* ≤ 0.05). Despite an improvement in the detection of shorter signal durations as a function of retest session, HIV-1 Tg animals failed to improve as quickly or to the same magnitude as control animals. Data were extrapolated to 10 msec using linear regression calculations to demonstrate the point at which animals experiences a loss of signal detection (i.e., the intersection of hits and misses). A blue line indicates the point at which HIV-1 Tg animals experienced a loss of signal detection. Data are presented as mean ± SEM.

Signal Detection: Across retest assessments, genotype became the driving factor for observed differences in sustained attention. Independent of genotype, HIV-1 Tg and control animals exhibited a significant improvement in the detection of shorter signal durations across retest assessments (Fig. 4). However, HIV-1 Tg animals displayed a progressive, relative impairment in the detection of shorter signal durations relative to control animals (Genotype × Response Type × Duration × Retest Assessment Interaction [*F*(8,488) = 2.2, *p*_GG_ ≤ 0.05, η_p_^2^ = 0.034, Power = 0.763] with a prominent quadratic-quadratic component [*F*(1,61) = 7.3, *p* ≤ 0.009, η_p_^2^ = 0.106, Power = 0.756]). Specifically, HIV-1 Tg animals took significantly longer to reach the same level of performance (e.g., Loss of Signal Detection at 10 msec: Control animals (Retest 1); HIV-1 Tg animals (Retest 5)) and failed to improve to the level of control animals at any retest assessment.

Relative to genotype, the factor of biological sex played a less prominent role in the progression of sustained attention (Sex × Response Type × Duration × Retest Assessment Interaction with a prominent linear-linear component [*F*(1,61) = 5.3, *p* ≤ 0.025, η_p_^2^ = 0.080, Power = 0.622]). Female animals, independent of genotype, failed to improve to the same level of performance as male animals.

### Phase 3: 18 Month Assessment

#### A more challenging signal detection task at 18 months of age, in combination with a reversal task, tapping flexibility and inhibition, revealed marked impairment, dependent upon task and biological sex, in HIV-1 Tg rats relative to control animals

**Figure 5:**
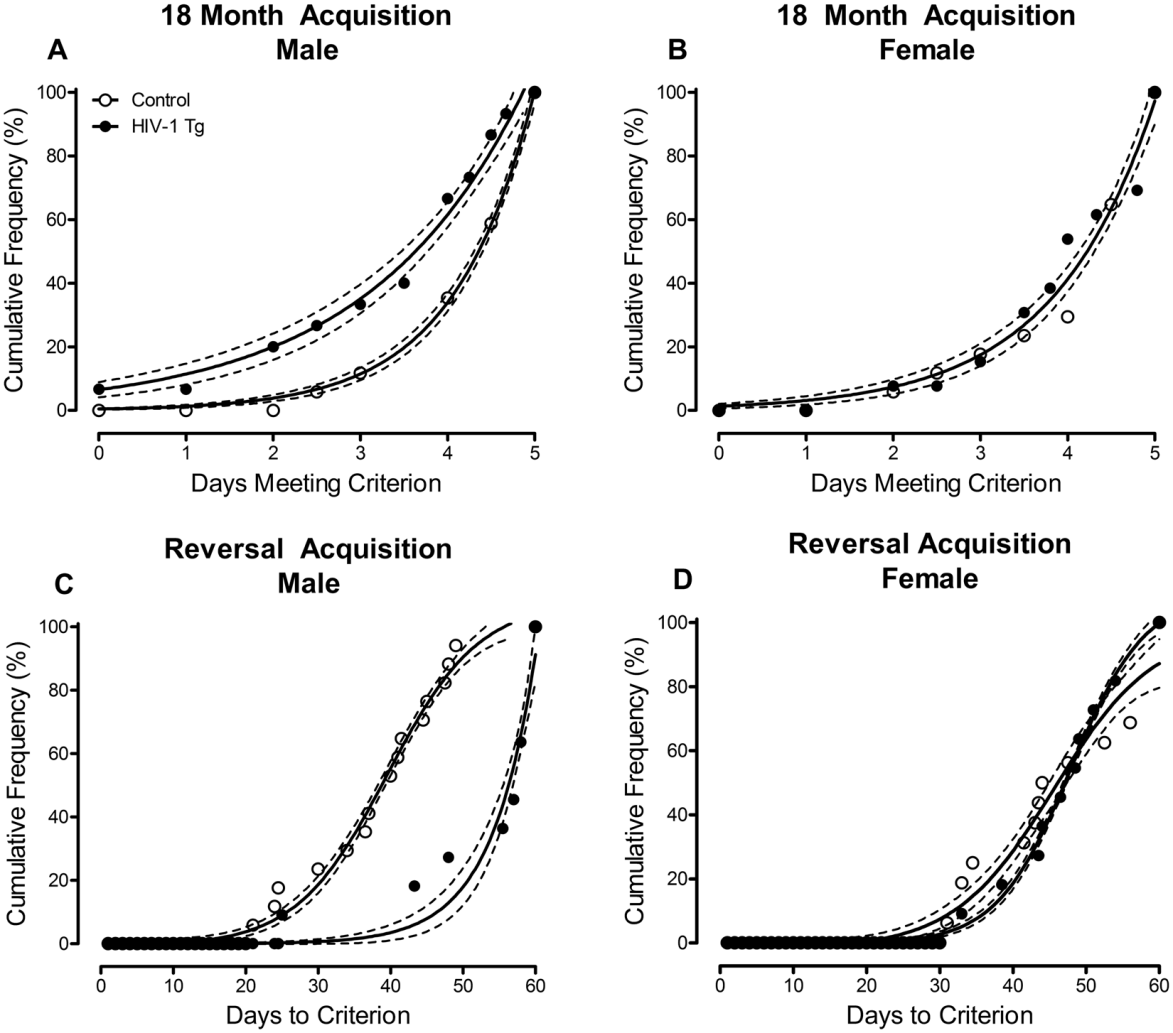
Temporal process of acquisition during the 18 month acquisition (**A**,**B**) and reversal (**C**,**D**) task. During the 18 month acquisition task, HIV-1 Tg and control animals were challenged for 5 consecutive days with shorter signal durations (i.e., 1000, 100, 10 msec). Male HIV-1 Tg animals (**A**) displayed a shift towards a decreased number of days meeting criteria (i.e., 70% accuracy) relative to male control animals (*p* ≤ 0.001); a distribution shift not observed in female HIV-1 Tg animals (**B**) *p* > 0.05). The temporal process of acquisition in the reversal task was assessed for HIV-1 Tg animals that had two months (i.e., 60 days) to acquire the task. Male HIV-1 Tg animals took significantly longer to acquire the reversal task relative to male control animals (**C**) *p* ≤ 0.005); an impairment not observed in female HIV-1 Tg animals (**D**) *p* > 0.05). Data are presented as cumulative frequencies with 95% confidence intervals fit to the curve.

Temporal Process of Acquisition: At 18 months of age, HIV-1 Tg and control animals were challenged for 5 consecutive days with shorter signal durations (i.e., 1000, 100, 10 msec). The number of days HIV-1 Tg and control animals met criterion was analyzed using a curve-fitting analysis. For male animals, independent of genotype, an exponential growth equation provided a well-described fit (Fig. 5A; *R*^*2*^*s* ≥ 0.98). However, significant differences in the parameters of the function were observed (*F*(2,15) = 57.4, *p* ≥ 0.001), indicating a shift in the distribution, with male HIV-1 Tg animals meeting criterion on fewer days relative to male control animals. In sharp contrast, a global, exponential growth equation (*R*^*2*^ ≥ 0.96) provided a well-described fit for female HIV-1 Tg and control animals (Fig. 5B), indicating no significant genotype differences in the number of days meeting criterion during the 18 month acquisition.

Subsequently, animals were assessed in a reversal task, tapping flexibility and inhibition. Presence of the HIV-1 transgene had a profound effect on the temporal process of acquisition in reversal, dependent upon the factor of biological sex (Fig. 5C,D; Genotype x Sex Interaction: [*F*(1,51) = 4.8, *p* ≤ 0.033]).

Complementary analyses were conducted for each sex to determine the locus of the interaction. In male animals (Fig. 5C), presence of the HIV-1 transgene significantly altered the temporal process of acquisition with male HIV-1 Tg animals acquiring the task significantly slower than male control animals (Main Effect, Genotype: [*F*(1,26) = 9.5, *p* ≤ 0.005]). In sharp contrast, presence of the HIV-1 transgene failed to significantly alter the temporal process of acquisition in female animals (Fig. 5D; p > 0.05).

Signal Detection: Assessment of signal detection data revealed a significant Task × Response Type × Duration × Genotype × Sex interaction [*F*(2,108) = 7.2, *p*_GG_ ≤ 0.002, η_p_^2^ = 0.117, Power = 0.886] with a prominent linear-quadratic component [*F*(1,54) = 10.0, *p* ≤ 0.003, η_p_^2^ = 0.156, Power = 0.874].

**Figure 6:**
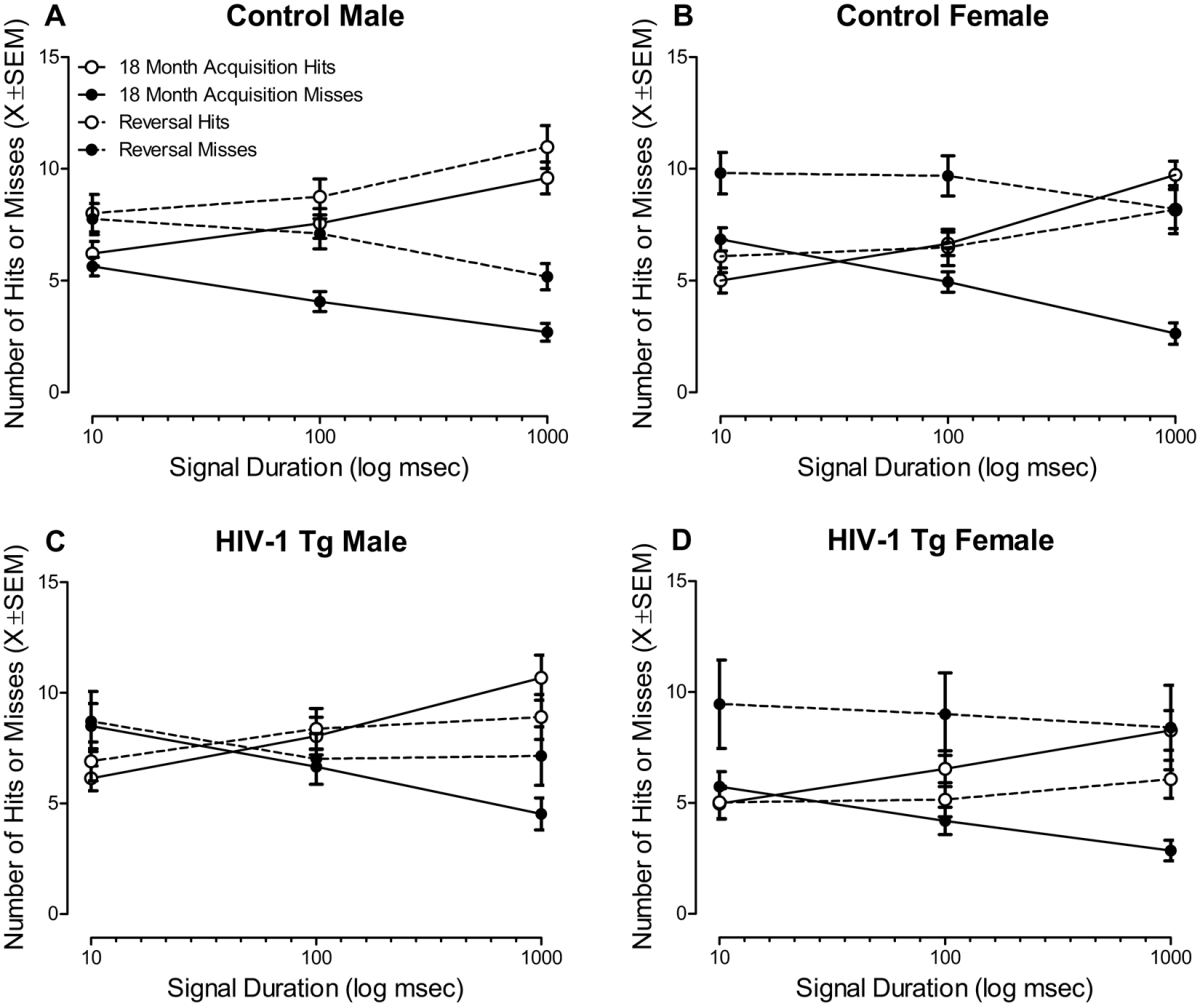
Signal detection data, presented as mean ± SEM, during the 18 month acquisition and reversal task are illustrated as a function of genotype and sex. A significant Task × Response Type × Genotype × Sex interaction [*F*(2, 108) = 7.2, *p*_GG_ ≤ 0.002, η_p_^2^ = 0.117] was observed. Male HIV-1 Tg (**C**) animals displayed a profound impairment in the detection of shorter signals during 18 month acquisition task and reversal task relative to male control (**A**) animals. Female HIV-1 Tg animals (**D**) failed to exhibit a deficit in the detection of shorter signals during the 18 month acquisition task relative to female control (**B**) animals. However, female HIV-1 Tg animals exhibited prominent impairments in flexibility and inhibition, evidenced by an inability to reliably detect the signal at any duration assessed during the reversal task.

Male HIV-1 Tg animals revealed marked impairment in signal detection relative to male control animals evidenced by a significant rightward shift in the loss of signal detection during the 18 month acquisition (Fig. 6A,C). During the reversal assessment, male control animals displayed a significant increase in both the number of hits and misses, suggesting an overtraining reversal effect. No significant alterations in the number of hits and/or misses was observed in the male HIV-1 Tg animals, suggesting that, despite extensive training, there was no improvement in their performance during the reversal task.

In sharp contrast, at 18 months of age, female HIV-1 Tg animals displayed no impairment in the detection of shorter signal durations relative to female control animals (Fig. 6B,D). However, female HIV-1 Tg animals, relative to female control animals, exhibited marked impairment in the reversal task evidenced by an inability to reliably detect the signal at any duration assessed.

### Phase 4: Neuroanatomical Assessments

#### Selective alterations in neuronal morphology were observed in pyramidal neurons from layers II-III of the medial prefrontal cortex in HIV-1 Tg animals, suggesting an alteration in dendritic branching complexity, but not neuronal arbor complexity or dendrite length

**Figure 7:**
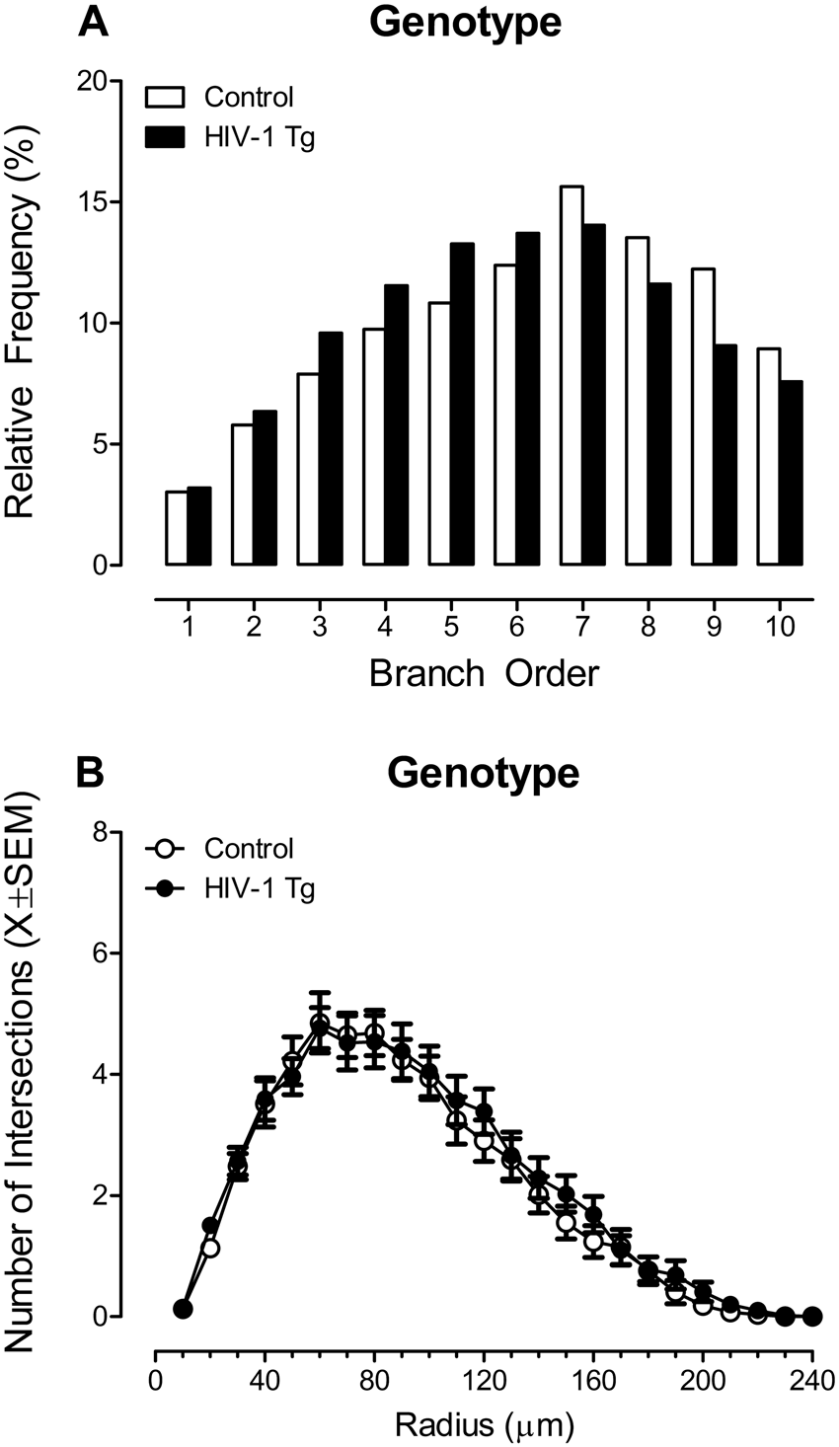
Two assessments of neuronal morphology, including a branch order analysis (**A**) and Sholl analysis (**B**) were conducted in pyramidal neurons from the medial prefrontal cortex. Branch order analyses (**A**) revealed an increased relative frequency of dendritic branches at lower branch orders in HIV-1 Tg animals relative to controls. In sharp contrast, neither presence of the HIV-1 transgene nor the factor of biological sex influenced the number of dendritic intersections occurring every 10 µm from the soma, assessed using a Sholl analysis (**B**). Thus, HIV-1 Tg animals exhibited selective alterations in the morphology of pyramidal neurons from layers II-III of the mPFC, characterized by decreased dendritic branching complexity. Data are illustrated as relative frequencies of the entire dataset (**A**) or mean ± SEM (**B**).

Branch Order: A centrifugal branch ordering method was utilized in Neurolucida 360 to automatically assign each dendrite with a branch order by counting the number of segments traversed. Presence of the HIV-1 transgene had a profound effect on the number of dendritic branches at each branch order (Fig. 7A; Genotype x Branch Order Interaction [*F*(1,952) = 10.1, *p* ≤ 0.002]). Specifically, HIV-1 Tg animals exhibited an increased relative frequency of dendritic branches at lower branch orders relative to controls, suggesting an alteration in dendritic branching complexity.

Sholl Analyses: Subsequently, a Sholl analysis was conducted as a complementary measure of neuronal arbor complexity, whereby the number of dendritic intersections occurring every 10 µm from the soma were quantified in HIV-1 Tg and control animals. Neither presence of the HIV-1 transgene nor the factor of biological sex influenced the number of intersections at successive radii (Fig. 7B; *p* > 0.05) or the total dendrite length (*p* > 0.05; Control Male: 729.3 ± 83.1 µm, Control Female: 740.6 ± 49.7 µm; HIV-1 Tg Male: 679.1 ± 81.0 µm; HIV-1 Tg Female: 758.4 ± 42.5 µm). Thus, HIV-1 Tg animals exhibited selective alterations in the morphology of pyramidal neurons from layers II-III of the mPFC, characterized by decreased dendritic branching complexity.

#### HIV-1 Tg animals displayed a profound shift in the distribution of dendritic spines in pyramidal neurons from layers II-III of the medial prefrontal cortex, supporting an alteration in synaptic connectivity

**Figure 8:**
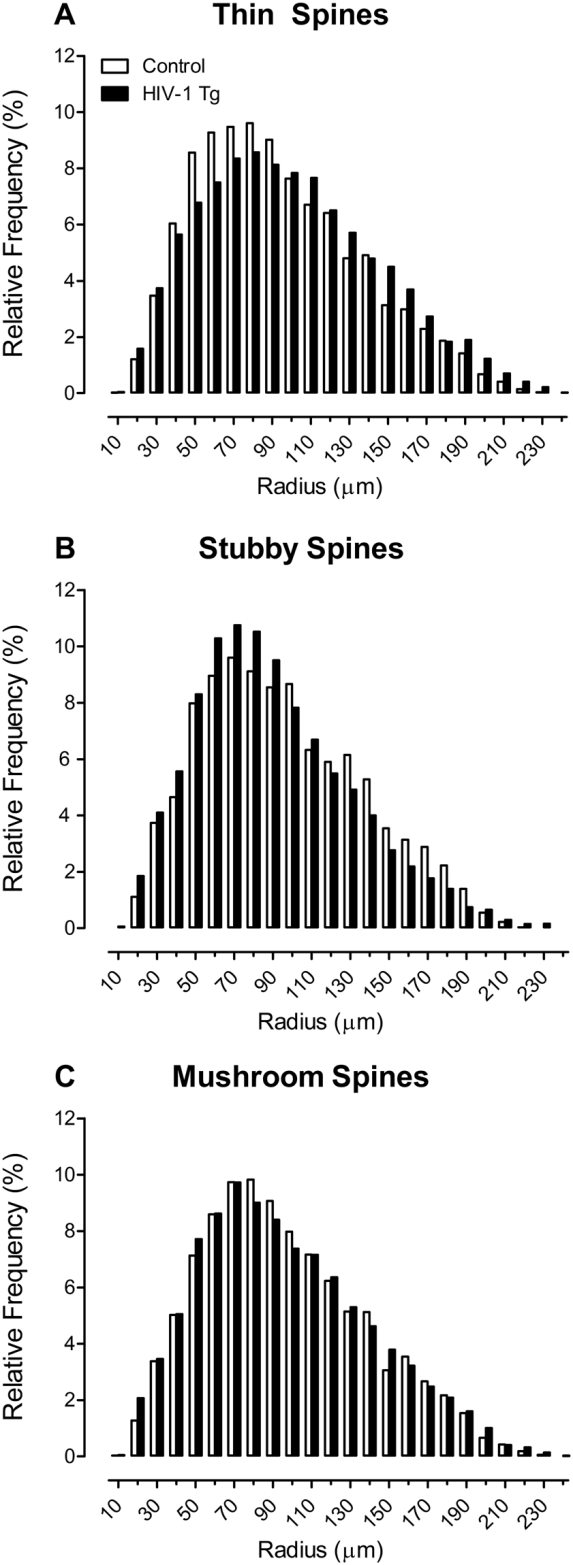
The distribution of dendritic spines on pyramidal neurons from the medial prefrontal cortex are illustrated as a function of spine type (i.e., Thin: (**A**), Stubby: (**B**), Mushroom: (**C**)), genotype (HIV-1 Tg vs. control) and radii. HIV-1 Tg animals exhibited a prominent distributional shift in the relative frequency of thin spines (**A**), with an increased relative frequency on more distal branches, relative to controls. In sharp contrast, HIV-1 Tg animals displayed an increased relative frequency of stubby spines (**B**) on more proximal branches, relative to controls. No genotypic alterations were observed in the distribution of mushroom spines (**C**). Data are illustrated as relative frequencies of the entire dataset.

Sholl analyses were subsequently utilized to determine where dendritic spines were located on the neuron, assessed using spine type (i.e., Thin Spines, Stubby Spines, Mushroom Spines), determined using an observer-assisted automatic classification system in Neurolucida 360, genotype (i.e., HIV-1 Tg, Control), biological sex (i.e., Male, Female) and radii as factors (Fig. 8). HIV-1 Tg animals exhibited a prominent shift in the distribution of dendritic spines dependent upon the factor of biological sex and spine type (Genotype × Sex × Spine Type × Radii Interaction [*F*(2, 6265) = 21.5, *p* ≤ 0.001]). The effect or lack of effect of biological sex on spine type is illustrated in Supplementary Fig. 2.

Complementary analyses were conducted on each spine type to determine the locus of these interactions. HIV-1 Tg animals displayed a prominent shift, with an increased relative frequency of thin dendritic spines on more distal branches relative to control animals (Genotype × Radii Interaction [*F*(1,2057) = 83.1, *p* ≤ 0.001]); the magnitude of which was influenced by the factor of biological sex (Genotype × Sex × Radii Interaction [*F*(1,2057) = 19.1, *p* ≤ 0.001]). In sharp contrast, an assessment of stubby dendritic spines revealed a population shift with an increased relative frequency on more proximal branches in HIV-1 Tg animals relative to controls (Genotype × Radii Interaction [*F*(1,2057) = 14.1, *p* ≤ 0.001]); an effect influenced by the factor of biological sex (Genotype × Sex × Radii Interaction [*F*(1,2057) = 27.3, *p* ≤ 0.001]), whereby the effect was observed in female HIV-1 Tg animals (Genotype x Radii Interaction [*F*(1,957) = 50.9, *p* ≤ 0.001]), but not male HIV-1 Tg animals (Genotype × Radii Interaction: *p* > 0.05). No significant differences in the distribution of mushroom dendritic spines were observed (*p* > 0.05).

#### A selective population shift in dendritic spine morphology was observed in layers II-III pyramidal neurons of the medial prefrontal cortex dependent upon presence of the HIV-1 transgene and biological sex

Measurement of dendritic spine parameters, including backbone length, head diameter, and volume (see Supplementary Fig. 3) revealed a selective population shift in dendritic spine morphology in layers II-III pyramidal neurons of the mPFC dependent upon presence of the HIV-1 transgene and biological sex. Male HIV-1 Tg animals exhibited a population shift towards shorter dendritic spines with no observed alterations in head diameter or volume. In sharp contrast, female HIV-1 Tg animals displayed a population shift towards longer dendritic spines with decreased head diameter relative to female control animals; no significant alterations in dendritic spine volume were revealed in female HIV-1 Tg animals relative to female control animals. A generalized linear mixed effects model with a Poisson distribution confirmed these observations, revealing a significant Genotype × Sex × Bin interaction for both backbone length [*F*(1,1659) = 37.2, *p* ≤ 0.001] and head diameter [*F*(1,1154) = 68.2, *p* ≤ 0.001]; an effect not observed for dendritic spine volume (*p* > 0.05).

#### There was no significant difference in neuroinflammation in the hippocampus between HIV-1 Tg and control animals

**Figure 9:**
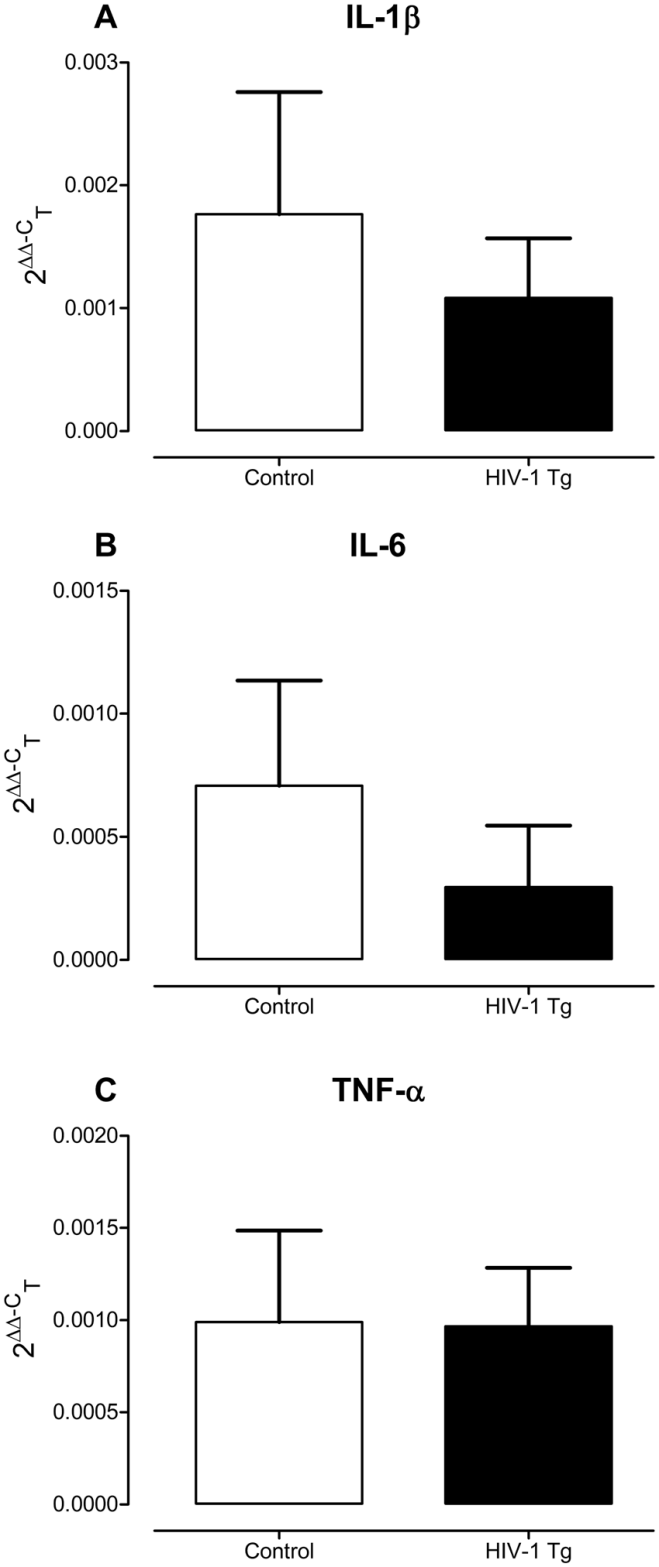
Three neuroinflammatory markers, including IL-1β (**A**), IL-6 (**B**), and TNF-α (**C**), were assessed in the hippocampus of HIV-1 Tg and control animals. Overall, HIV-1 Tg and control animals, independent of biological sex, reaching threshold after 40 cycles, displayed low levels of gene expression; levels which were independent of genotype or sex (*p* > 0.05). Data are presented as mean ± SEM.

Three putative neuroinflammatory markers, including IL-1β, IL-6, and TNF-α, were assessed in the hippocampus of HIV-1 Tg and control animals (Fig. 9). Overall, HIV-1 Tg and control animals, independent of biological sex, reaching threshold after 40 cycles, displayed low levels of gene expression. No significant genotype (*p* > 0.05) or sex (*p* > 0.05) differences in gene expression, examined using the 2^−ΔΔCt^ method, were observed.

### Phase 5: Mechanistic Basis of Neurocognitive Impairment

#### Neurocognitive impairments across the functional lifespan explain significant genotypic variance

**Figure 10:**
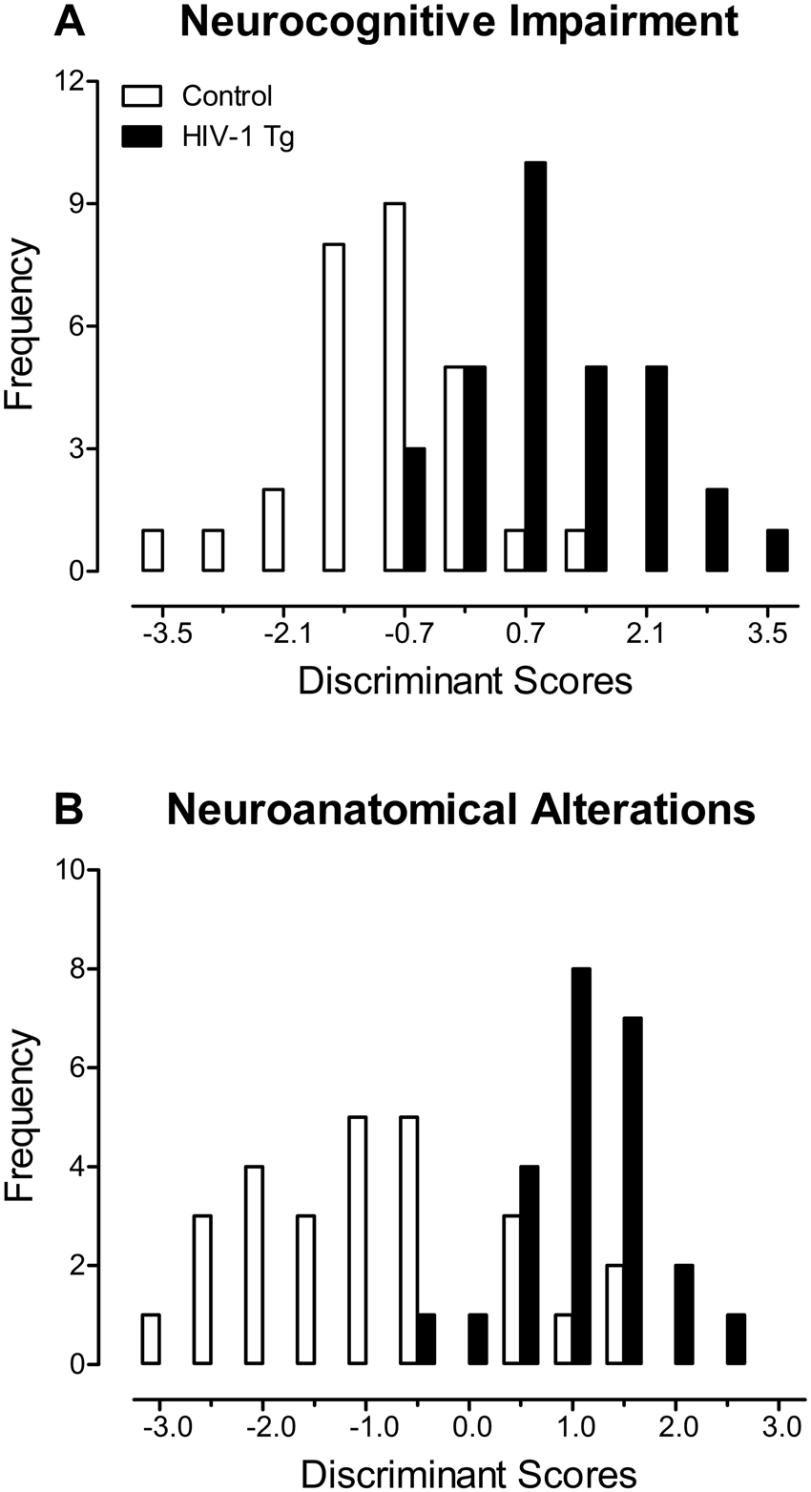
A discriminant function analysis was utilized to determine whether neurocognitive impairments (**A**) or synaptic dysfunction in the medial prefrontal cortex (mPFC; **B**) could classify animals based on genotype. Seven variables, corresponding to alterations in learning and sustained attention, correctly classified the presence of the HIV-1 transgene with 78.5% accuracy, accounting for 61.6% of the genotypic variance in neurocognitive function (**A**). Seven variables, corresponding to alterations in dendritic spine connectivity and dendritic spine head diameter, correctly classified the presence of the HIV-1 transgene with 80.4% accuracy, accounting for 64.6% of the genotypic variance in synaptic dysfunction in the mPFC (**B**).

A discriminant function analysis (DFA) was utilized to determine whether neurocognitive assessments conducted across the functional lifespan could classify animals based on genotype (Fig. 10A). Variables included in the stepwise DFA represented alterations in sustained attention (Hits and Misses at 1000, 500, and 100 msec during Original Acquisition and Retest 1–5; Hits and Misses at 1000, 100, and 10 msec during the 18 Month Assessment), learning (Days to Criteria during Original Acquisition, Days to Criteria at Retest 1–5, Number of Days Meeting Criteria during the 18 Month Assessment), and flexibility and inhibition (Hits and Misses at 1000, 100, and 10 msec during the Reversal Assessment).

HIV-1 Tg and control animals were maximally separated (canonical correlation of 0.70) by selecting seven variables corresponding to sustained attention (i.e.,Hits at 1000 msec during Original Acquisition, Hits at 500 msec during Retest 1, Misses at 1000 msec during Retest 2, Misses at 100 msec during Retest 4, Hits at 1000 msec during Retest 5, and Misses at 10 msec during the 18 Month Assessment) and learning (i.e., Days to Criteria during Retest 5). Animals were correctly classified for presence of the HIV-1 transgene (jack-knifed classification) with 78.5% accuracy (Approximately of Wilks’ λ of 0.516, χ^2^(7) = 39.4, *p* ≤ 0.001), accounting for approximately 61.6% of the genotypic variance.

#### Synaptic dysfunction, assessed in pyramidal neurons from layers II-III of the medial prefrontal cortex, explains significant genotypic variance

A DFA was utilized to determine whether synaptic dysfunction in pyramidal neurons from layers II-III of the mPFC could classify animals based on genotype (Fig. 10B). Variables included in the stepwise DFA represented alterations in dendritic branching complexity, dendritic spine connectivity, and dendritic spine morphology.

HIV-1 Tg and control animals were maximally separated (canonical correlation of 0.72) by selecting seven variables corresponding to dendritic spine connectivity (i.e., Relative Frequency of Thin Dendritic Spines at 150 µm; Relative Frequency of Stubby Dendritic Spines at 40 µm, 110 µm, and 170 µm) and dendritic spine head diameter (i.e., Relative Frequency of Dendritic Spines within the 0.075 µm, 0.225 µm and 0.375 µm bin). Animals were correctly classified for presence of the HIV-1 transgene (jack-knifed classification) with 80.4% accuracy (Approximately of Wilks’ λ of 0.489, χ^2^(7) = 32.5, *p* ≤ 0.001), accounting for approximately 64.6% of the genotypic variance.

## Discussion

Progressive NCI, including alterations in learning, sustained attention, flexibility, and inhibition, were observed in the population of HIV-1 Tg rats sampled. During initial acquisition, biological sex was the driving factor for observed differences in learning and sustained attention. Across retest assessments, presence of the HIV-1 transgene became the prominent factor underlying alterations in the temporal process of task acquisition, as well as sustained attention. Nevertheless, at 18 months of age, sex-dependent expression of NCI were observed in HIV-1 Tg animals, relative to controls. Pyramidal neurons from layers II-III of the mPFC revealed profound synaptic dysfunction in HIV-1 Tg animals relative to controls; dysfunction that was characterized by alterations in dendritic branching complexity, synaptic connectivity, and dendritic spine morphology. NCI across multiple domains (i.e., learning, sustained attention) and synaptic dysfunction in the mPFC independently identified the presence of the HIV-1 transgene with at least 78.5% accuracy, accounting for at least 61.6% of the genotypic variance. Thus, even in the absence of sensory or motor system deficits^14^ and comorbidities, HAND is a neurodegenerative disease characterized by age-related disease progression; impairments which may be due, at least partly, to synaptic dysfunction in the mPFC.

Located at the anterior pole of the mammalian brain, the prefrontal cortex (PFC), recognized as an anatomically complex brain region^35^, is organized in a laminar fashion and comprised of three major subdivisions (i.e., mPFC, orbital PFC (oPFC) and lateral PFC). Executive functions, including attention, flexibility, and inhibition, are the primary, most basic function of the PFC^36^. Although executive functions cannot be localized to any single subdivision of the PFC, the mPFC, oPFC, and lateral PFC each exhibit some degree of functional specialization^35,36^. Specifically, the mPFC has been implicated as having a primary role in attention, including pre-attentive processes [e.g.^37^] and sustained attention [e.g.^38^], whereas the oPFC is involved in stimulus-reinforcement learning [e.g.^39^] and reversal [i.e., flexibility and inhibition^40^].

The utilization of a series of neurocognitive tasks across the functional lifespan tapped multiple subdivisions of the PFC, revealing progressive NCI in the HIV-1 Tg rat. At the genotypic level, HIV-1 Tg animals displayed a progressive, relative impairment in tasks tapping both the mPFC (i.e., sustained attention) and the oPFC (i.e., stimulus-reinforcement learning). Most notably, HIV-1 Tg animals also exhibited alterations in the progression of prepulse inhibition, a pre-attentive process tapping, in part, the mPFC^14^. However, sex differences in the presentation of NCI at 18 months of age were dependent upon the brain region tapped via neurocognitive assessments (i.e., mPFC vs. oPFC). Specifically, male HIV-1 Tg animals exhibited significantly greater impairment, relative to male control animals, in sustained attention and stimulus-reinforcement learning in the reversal assessment, tasks tapping both the mPFC and oPFC. Female HIV-1 Tg animals, however, displayed profound impairment, relative to female control animals, in flexibility and inhibition, tasks predominantly associated with the oPFC. Elucidating the mechanisms underlying sex-dependent expression of NCI is vital for the development of therapeutic treatments and cure strategies.

The rich cortical and subcortical connections make the PFC ideally positioned to serve as a central hub, integrating and relaying information from multiple afferent sources^41^. Broadly, the PFC receives afferents from thalamic nuclei, the limbic system, the frontal cortex, and other brain regions [e.g., hypothalamus, cerebellum, mesencephalon^36^]. Dense innervation from multiple neurotransmitter systems to the PFC has also been well-established^36^; afferents which play a prominent regulatory role across the PFC^36^ and are functionally involved in the control of executive functions [for review^42^]. Specifically, noradrenergic projections from the locus coeruleus (LC), dopaminergic afferents from the ventral tegmental area (VTA), and serotonergic projections from the raphe nuclei innervate the PFC.

Excitatory pyramidal neurons, characterized by a single apical dendrite, multiple shorter basal dendrites, and thousands of dendritic spines are abundant throughout the PFC^43^. A synaptic relationship between pyramidal neurons and the major afferent systems is established via postsynaptic sites on dendritic shafts and spines; the vast majority of which occur on dendritic spines [e.g.^44^]. Morphologically, dendritic spines are classically characterized into three primary categories [i.e., thin, stubby, mushroom^45^]. Thin spines, the predominant spine type in both HIV-1 Tg and control animals, are characterized by a long, thin neck and a small bulbous head, whereas the head volume to neck volume ratio is higher in mushroom spines. In sharp contrast, stubby spines are devoid of a spine neck, exhibiting an approximately equal head and neck volume ratio^45^. Most notably, asymmetric excitatory synapses are primarily formed between dendritic spine heads and presynaptic axons [e.g.^46^], however, some dendritic spines receive additional input on their neck^47^. Thus, morphological characteristics of dendritic spines, which are reflective of functionality and capacity for structural change^48^, and their distribution along the dendrite, may be associated with synaptic dysfunction in the HIV-1 Tg rat.

**Figure 11:**
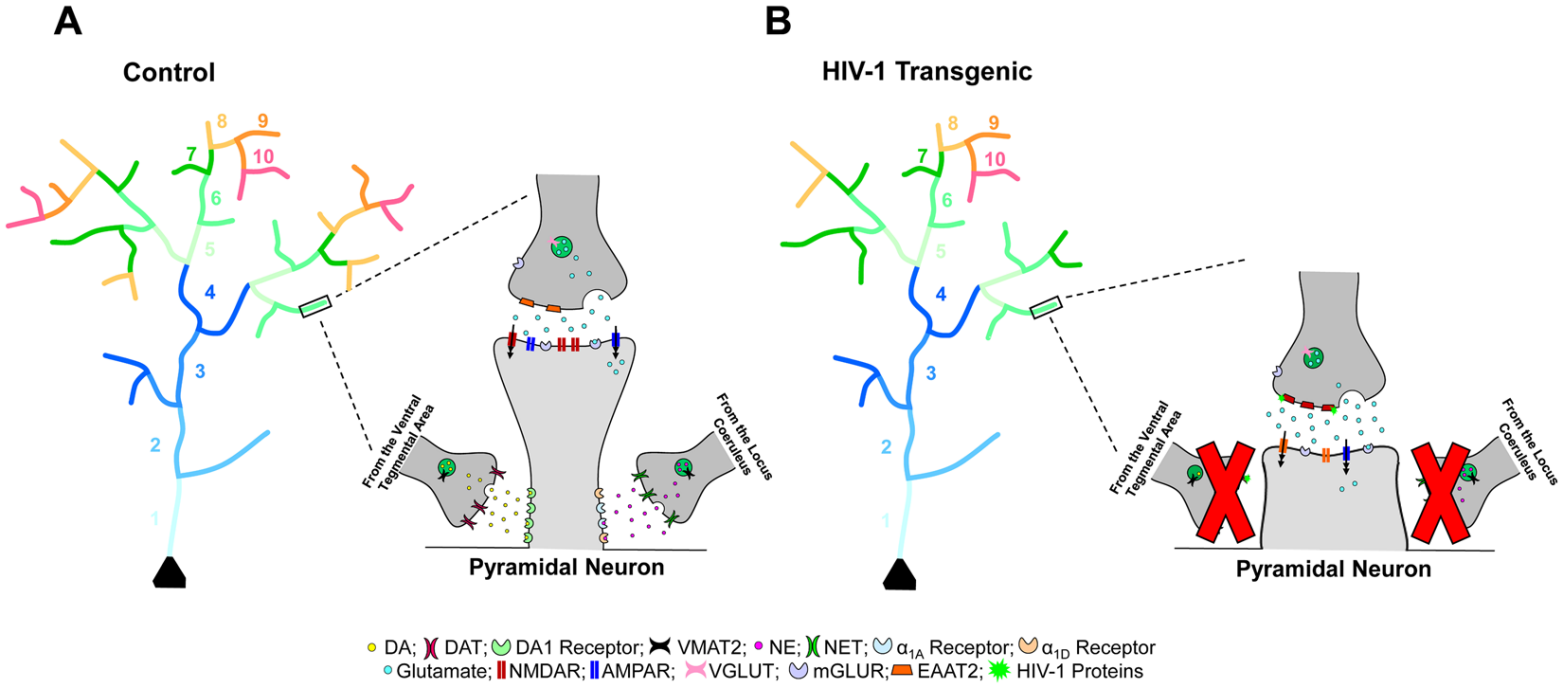
Dendritic spine connectivity in pyramidal neurons of the medial prefrontal cortex is illustrated as a function of genotype (HIV-1 Tg vs. control). Pyramidal neurons in HIV-1 Tg animals (**B**) displayed decreased dendritic branching complexity, with a decreased relative frequency of higher order branches relative to control animals (**A**) with no observed alterations in total dendrite length. Control animals exhibited a preponderance of thin spines on more proximal branches, receiving dopaminergic afferents from the ventral tegmental area (VTA) and noradrenergic innervation from the locus coeruleus (LC). In sharp contrast, HIV-1 Tg animals displayed a preponderance of stubby spines on more proximal branches. Morphological characteristics of stubby spines, including the absence of a spine neck^45^ and decreased postsynaptic density on the dendritic spine head^52^ suggest that HIV-1 Tg animals failed to receive appropriate dopaminergic afferents from the VTA and/or noradrenergic innervation from the LC. Illustrated here is the reception of dopaminergic and noradrenergic afferents on the dendritic spine neck, although other mechanisms are possible. Abbreviations: DA: Dopamine, DAT: Dopamine Transporter, DA1 Receptor: Dopamine Receptor D1, VMAT2: Vesicular Monoamine Transporter 2, NE: Norepinephrine, NET: Norepinephrine Transporter, α_1A_ Receptor: Alpha-1A Adrenergic Receptor, α_1D_ Receptor: Alpha-1D Adrenergic Receptor, NMDAR: NMDA Receptor, AMPAR: AMPA Receptor, VGLUT: Vesicular Glutamate Transporter, mGLUR: Metabotropic Glutamate Receptor, EAAT2: Excitatory Amino Acid Transporter 2.

Neuronal morphology, assessed using a branch order analysis, tapping dendritic branching complexity, as well as the classical Sholl analysis, tapping neuronal arbor complexity, revealed prominent alterations in HIV-1 Tg animals relative to controls (Fig. 11). In pyramidal neurons from layers II-III of the mPFC, assessed in the present study, HIV-1 Tg animals exhibited decreased dendritic branching complexity; an effect independent of biological sex. No statistically significant genotypic differences were observed in dendritic length, consistent with previous reports in younger HIV-1 Tg animals^49^ or neuronal arbor complexity. In sharp contrast, in medium spiny neurons (MSNs) from the nucleus accumbens core subregion (NAcc), published results suggest sex-dependent alterations in neuronal morphology^50^. Specifically, female HIV-1 Tg animals, but not male HIV-1 Tg animals, displayed profound alterations in dendritic branching complexity and neuronal arbor complexity^50^. Morphological differences between pyramidal neurons, characterized as polar neurons, and MSNs, characterized by a centrifugal morphology, may underlie differences in branch order and Sholl analyses. Overall, results support alterations in neuronal morphology in HIV-1 Tg rats independent of brain region assessed. However, the comparison of results from branch order analyses with those from Sholl analyses suggests that the two analytic measures may reflect different measures of neuronal morphology.

HIV-1 Tg animals, independent of biological sex, exhibited a profound shift in the distribution of dendritic spines along the apical dendrite, supporting a prominent alteration in synaptic connectivity. Specifically, a preponderance of thin spines were observed on more distal dendrites, extending into layer I of the mPFC, in HIV-1 Tg animals, relative to controls. However, HIV-1 Tg animals, relative to controls, displayed an increased relative frequency of stubby spines on more proximal dendrites; an effect which was most prominent in female HIV-1 Tg rats. Notably, pyramidal neurons within layers II-III of the mPFC express multiple monoamine receptors, including noradrenergic receptors (i.e., primarily α_1A_, α_1D_), dopaminergic receptors (i.e., primarily D_1_) and serotonergic receptors (i.e., primarily 5-HT_1A_, 5-HT_2A_); minimal, if any, receptor expression within layer I of the mPFC^51^. The preponderance of stubby spines, morphologically characterized by the absence of a dendritic spine neck^45^ and a smaller postsynaptic density^52^, on more proximal dendrites suggests that HIV-1 Tg animals fail to receive afferent projections, and thus neurotransmitter innervation, from the VTA, LC, and raphe nuclei (Fig. 11); consistent with neurotransmitter system alterations, specifically dopaminergic system dysfunction, commonly reported in HIV-1 [e.g.^53–56^]. One previous study^49^ and one conference abstract^57^ have also suggested alterations in dendritic spine density in the mPFC in younger HIV-1 Tg animals; an effect which may also influence synaptic connectivity. Thus, profound synaptic dysfunction was observed in HIV-1 Tg animals, characterized by alterations in dendritic branching complexity, synaptic connectivity (i.e., VTA-PFC-LC), and dendritic spine morphology, supporting a key neural mechanism for expression of NCI in HAND.

**Figure 12:**
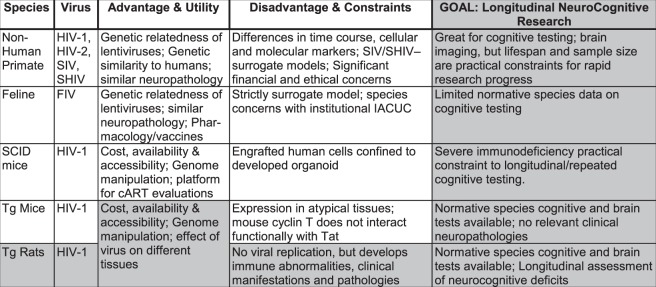
Several major considerations of the prominent animal species and their applicability to provide a biological system to model neuroHIV in the post-cART era are highlighted.

Multiple animal systems are available to model components of neuroHIV, each with their own advantages and constraints. Several major considerations of the prominent animal species and their applicability to provide a biological system to model neuroHIV in the post-cART era are highlighted in Fig. 12. Central nervous system (CNS) infection occurs within two weeks of HIV-1 infectivity in humans^58,59^, leading to NCI [e.g.^6,7^] and behavioral alterations [e.g., Apathy^60^; Depression^61^]. Selective NCI [e.g., selective attention appears relatively spared^15^] have been observed across multiple domains in prominent animals systems [e.g., HIV-1 Tg rat, pre-attentive processes^13^; learning and memory^16,17^; executive function^15^; SIV, spatial working memory^62^; Humanized Mice, memory^63^; Tat Transgenic Mice, spatial learning^64,65^, reversal learning^64^]. Examples of behavioral alterations, similar to those observed in HIV-1 seropositive individuals, include depressive-like behaviors [e.g., HIV-1 Tg rat^66^; Tat transgenic mice^67,68^] and motivational alterations [e.g., HIV-1 Tg rat^56^]. Furthermore, although generally understudied, prominent sex differences in the expression of NCI have been observed in HIV-1 seropositive individuals, with HIV-1 seropositive females displaying more severe deficits relative to HIV-1 seropositive males^69,70^. Sex differences in the expression of NCI have been translationally modeled in the HIV-1 Tg rat^18,71,72^, but inconsistent results have been reported in other animal systems [e.g., Tat transgenic mice^73^]. Given the increased life expectancy of HIV-1 seropositive individuals in the post-cART era, as well as the persistence of NCI, an *in vivo* biological system able to provide a longitudinal study of HIV-1 is critical. HIV-1 seropositive individuals display progressive NCI, with individuals exhibiting asymptomatic NCI at baseline having an increased risk (i.e., two to six times) of developing symptomatic HAND^74^. The HIV-1 Tg rat, as demonstrated in the present study, displays age-related disease progression in multiple neurocognitive domains across the functional lifespan. Thus, the present study further elucidates the advantages and constraints of the HIV-1 Tg rat biological system to model neuroHIV in the post-cART era.

Despite the aforementioned strengths of the present study, a few caveats are acknowledged. First, the neuronal analysis focused exclusively on pyramidal neurons from layers II-III of the mPFC. Although the generalizability of synaptic dysfunction cannot be directly assessed within the present study, observations across multiple brain regions [i.e., nucleus accumbens, mPFC^12,21,49,50^], ages [i.e., 4 months; 14–17 months; 20 months^12,21,50^] and in HIV-1 Tg rats following psychostimulant exposure [i.e., methylphenidate^21^] suggests the importance of further studies investigating synaptic dysfunction as a neural mechanism underlying NCI in HAND. Second, neuroinflammatory markers were assessed exclusively in the hippocampus and using only three cytokines (i.e., IL-1β, IL-6, and TNF-α). Despite the utilization of two experimental methodologies and sufficient statistical power, no significant differences in neuroinflammation in the hippocampus were suggested between HIV-1 Tg and control animals; results consistent with those observed using [(18)F]-DPA714 PET imaging and a profile of 24 neuroinflammatory markers in (3 and 9 month) adult HIV-1 Tg animals^75^ and following PD 1 stereotaxic injections of HIV-1 viral proteins [i.e., Tat_1–86_, gp120^29^]. However, this does not preclude neuroinflammation in the adolescent brain, in other brain regions, or using other methodology [e.g., ELISA^76^]. For example, results of the present study are in contrast to those reported by Royal *et al*.^76^, which observed significant increases in the expression of INF-γ, TNF-α, and IL-β, assessed using ELISA, in brain lysates prepared from the frontal cortex and subcortical white matter of the HIV-1 Tg rat.

Even in the absence of sensory or motor system deficits^14^ and comorbidities, HAND is a neurodegenerative disease. NCI are characterized by age-related disease progression across multiple neurocognitive domains, tapping two primary subdivisions of the PFC. Most notably, alterations in sustained attention and learning across the functional lifespan, account for approximately 61.6% of the genotypic variance in NCI, independent of biological sex. Synaptic dysfunction in pyramidal neurons from layers II-III of the mPFC, characterized by alterations in dendritic branching complexity, synaptic connectivity, and dendritic spine morphology, supports a key, albeit not exclusive, neural mechanism underlying HAND, accounting for approximately 64.6% of the genotypic variance. Thus, the progression of HAND with age in the HIV-1 Tg rat and the associated synaptic dysfunction affords an instrumental model system for the development of therapeutics and functional cure strategies.

## Supplementary information

Supplementary Material

## Data Availability

All relevant data are within the paper.
