# Supplementary material for: Disruption of Timing: NeuroHIV Progression in the Post-cART Era
Source: Sci Rep. 2019 Jan 29;9:827. doi: 10.1038/s41598-018-36822-1 (PMC6351586; doi:10.1038/s41598-018-36822-1)
Supplement: Supplementary file 1 — Supplementary Material [file 41598_2018_36822_MOESM1_ESM.docx]

**Disruption of Timing: NeuroHIV Progression in the Post-cART Era**

Kristen A. McLaurin, Hailong Li, Rosemarie M. Booze, Charles F. Mactutus*

Program in Behavioral Neuroscience

Department of Psychology

Barnwell College

1512 Pendleton Street

University of South Carolina

Columbia, SC 29208

Address proofs and correspondence to:

Charles F. Mactutus, Ph.D.

Department of Psychology

1512 Pendleton Street

University of South Carolina

Columbia, SC 29208

PH: +1 (803) 777-4137

FAX: +1 (803) 777-9558

E-mail: [mactutus@mailbox.sc.edu](mailto:mactutus@mailbox.sc.edu)

**SUPPLEMENTARY METHODS**

**Phase 1: Original Acquisition**

***Signal Detection***

The signal detection test sessions, which began with a 5-minute habituation period, were conducted during the light-cycle in a darkened operant chamber. After each trial began, two retractable levers were extended for 2 sec and remained extended for 6 sec for the animal to make a response. A variable intertrial interval (ITI) of 9±3 sec was present, during which time the levers were retracted. Approximately half of the animals were trained to press the left lever during signal trials and the right lever during non-signal trials (hits and correct rejections, respectively). The other half of the subjects were trained using the reverse set of rules. A correct response, including hits and correct rejections, was rewarded with a sucrose pellet. Incorrect responses, including misses and false alarms, were not rewarded. Animals were trained on each vigilance program until meeting criteria of at least 70% accuracy for 5 consecutive or 7 non-consecutive test sessions, at which point they were promoted to the next program. Percent accuracy was calculated as follows: (Total Number of Hits and Correct Rejections)/(Total Number of Correct and Incorrect Responses x 100).

**Statistical Analysis**

The temporal process of learning (i.e., the number of days to meet criterion) during original acquisition, as a function of retest assessment, and during reversal acquisition was analyzed using a generalized linear mixed effects model with a Poisson distribution and an unstructured covariance pattern (SAS/STAT Software 9.4, SAS Institute, Inc., Cary, NC). Genotype (HIV-1 Tg vs. control) and sex (male vs. female) were included as between-subject’s factors. For reversal acquisition, only the animals with at least 60 days to complete the task were included in the figures and analysis (Control: *N*=17 litters; male, *n*=24, female, *n*=24; HIV-1 Tg: *N*=18 litters; male *n*­=17; female, *n*=21).

The number of days meeting criterion during 18 month acquisition, to directly assess functional form, was analyzed using a curve-fitting analysis and fit with a 95% confidence interval (CI; GraphPad Software, Inc., La Jolla, CA, USA).

Signal duration data during original acquisition, as a function of retest assessment, during the 18 month acquisition, and the reversal task was analyzed using a mixed-factor ANOVA (SPSS Statistics 24, IBM Corp., Somer, NY) with genotype (HIV-1 Tg vs. control) and sex (male vs. female) serving as between-subject’s factors. Response type (hits vs. misses), signal duration (e.g., 1000, 500, 100 msec) and retest assessment served as the within-subject’s factors, as appropriate. During original acquisition and retest assessments, signal detection data was analyzed for the first 5 consecutive or 7 non-consecutive days that an animal achieved 70% accuracy. For the 18-month acquisition assessment, all 5 days were included in the analysis. For the reversal task, when an animal met criteria, the first 5 consecutive or 7 non-consecutive days that an animal achieved 70% accuracy were included. In the reversal assessment, if an animal failed to meet criteria, but had 60 days or had completed 45 days by approximately 20 months of age, the 7 days with the highest percent accuracy were included in the analysis. To illustrate the profound genotype differences in the progression of sustained attention across retest assessments, data in Figure 4 were statistically extrapolated to 10 msec; the 10 msec point was not included in the statistical analysis. The factor of retest assessment was included in the statistical analysis of the 18-month challenge and reversal task, thus only the animals that completed both assessments were included in the statistical analysis; figures represent all animals that completed a given assessment.

Censored data, either due to euthanization, an equipment malfunction, or failure to complete the retest assessment was handled using the mean series imputation method (Retest Assessment 3: HIV-1 Tg female, *n*=2; Retest Assessment 4: control female, *n*=1; Retest Assessment 5: control female, *n*=1, control male, *n*=1, HIV-1 Tg female, *n*=4, HIV-1 Tg male, *n*=2; 18 Month Challenge: control female, *n*=1, control male, *n*=1, HIV-1 Tg female, *n*=1; Reversal Assessment: control male, *n*=3, HIV-1 Tg female, *n*=1).

Neuronal morphology was assessed using two measures, including the number of segments at each branch order and the number of intersections at successive radii (i.e., Sholl analysis). The number of segments at each branch order were analyzed using a generalized linear mixed effects model with a Poisson distribution and an unstructured covariance pattern (SAS/STAT Software 9.4, SAS Institute, Inc., Cary, NC) with genotype (HIV-1 Tg vs. control), sex (male vs. female) and branch order (Branches 1 – 10) included as factors in the analysis. The number of intersections at successive radii was analyzed using a mixed-design ANOVA, restricted maximum likelihood estimation model parameters, a variance components covariance structure [77], and a random intercept and random slope (i.e., Radius; SAS/STAT Software 9.4, SAS Institute, Inc., Cary, NC).

Dendritic spine connectivity was evaluated using the number of dendritic spines, dependent upon spine type (i.e., thin, stubby, mushroom), between each radii. Dendritic spine backbone length, head diameter, and volume were examined as assessments of dendritic spine morphology. A generalized linear mixed effects model with a Poisson distribution and an unstructured covariance pattern was conducted using PROC GLIMMIX (SAS/STAT Software 9.4, SAS Institute, Inc., Cary, NC) for both dendritic spine connectivity and dendritic spine morphology. Analyses were conducted on the number of dendritic spines between each successive radii or the number of dendritic spines within each bin. Between-subject’s factors included genotype (HIV-1 Tg vs. Control) and biological sex (male vs. female).

The gene expression of each neuroinflammatory marker was analyzed individually using a two-way ANOVA, with genotype (HIV-1 Tg vs. control) and biological sex (male vs. female), serving as the between-subject’s factors.

A discriminant function analysis (DFA) was utilized to examine the utility of neurocognitive impairments across the lifespan or dendritic spine alterations to correctly classify animals based on their genotype (SPSS Statistics 24, IBM Corp., Somer, NY).

**SUPPLEMENTARY FIGURE 1**

**Supplementary Figure S1**. Temporal process of acquisition as a function of retest session (*N*=17-20 litters per genotype). At each individual retest session (i.e., Retest 1: **A**, Retest 2: **B**, Retest 3: **C**, Retest 4: **D**, Retest 5: **E**) HIV-1 Tg animals, independent of biological sex, took a significantly greater number of days to acquire the signal detection task (*p*≤0.05); an impairment which progressed as a function of retest session. Data are presented as cumulative frequencies with 95% confidence intervals fit to the curve.

**SUPPLEMENTARY FIGURE 2**

Supplementary Figure S2. The distribution of dendritic spines on successive radii is illustrated as a function of spine type (i.e., Thin Spines (A,B), Stubby Spines (C,D), and Mushroom Spines (E,F)) and biological sex (i.e., Male (A,C,E), Female (B,D,F)) (*N=*16-19 litters). HIV-1 Tg animals exhibited an increased relative frequency of thin dendritic spines on more distal branches relative to controls; the magnitude of which was influenced by the factor of biological sex. In sharp contrast, an assessment of stubby dendritic spines revealed a population shift with an increased relative frequency on more proximal branches in HIV-1 Tg animals relative to controls; an effect influenced by the factor of biological sex. No significant differences in the distribution of mushroom dendritic spines were observed.

**SUPPLEMENTARY FIGURE 3**

**Supplementary Figure S3.** Dendritic spine morphology. Presence of the HIV-1 transgene and the factor of biological sex influenced dendritic spine morphology. Male HIV-1 Tg animals exhibited a distributional shift towards shorter dendritic spines (**A**) relative to male control animals; no significant alterations in dendritic spine head diameter (**C**) or volume (**E**) were observed in male HIV-1 Tg animals. Female HIV-1 Tg animals displayed a distributional shift towards longer dendritic spines (**B**) with increased head diameter (**D**) relative to female control animals; no significant alterations in dendritic spine volume (**F**) were observed in female HIV-1 Tg animals. A generalized linear mixed effects model with a Poisson distribution confirmed these observations, revealing a significant genotype x sex x bin interaction for both backbone length [*F*(1,1659)=37.2, *p*≤0.001] and head diameter [*F*(1,1154)=68.2, *p*≤0.001]; an effect not observed for dendritic spine volume (*p*>0.05). Data are illustrated as relative frequencies.

**SUPPLEMENTARY TABLE 1**

Supplementary Table S1. Primers for Neuroinflammatory Markers

| Rat genes | Forward (5’-3’) | Reverse (5’-3’) | GenBank Identifiers |
| --- | --- | --- | --- |
| TNF-α | ACCACGCTCTTCTGTCTACTG | CTTGGTGGTTTGCTACGAC | NM 013693.3 |
| IL-1β | GCAATGGTCGGGACATAGTT | AGACCTGACTTGGCAGAGGA | NM 031512.2 |
| IL-6 | GCCCTTGCTGGTGGATGTT | GAGAGGGAGTGCTGCTTGGA | NM 010559.3 |
| β-Actin | AAGTCCCTCACCCTCCCAAAAG | AAGCAATGCTGTCACCTTCCC | NM 007393.5 |
